# Supplementary material for: Changes in geometrical aspects of a simple model of cilia synchronization control the dynamical state, a possible mechanism for switching of swimming gaits in microswimmers
Source: PLoS One. 2021 Apr 8;16(4):e0249060. doi: 10.1371/journal.pone.0249060 (PMC8031381; doi:10.1371/journal.pone.0249060)
Supplement: S1 File — (PDF) [file pone.0249060.s001.pdf]

Supplementary Information for:
Changes in geometrical aspects of a simple model of cilia synchronization control the dynamical state, a possible mechanism for switching of swimming gaits in microswimmers

Evelyn Hamilton[1] and Pietro Cicuta[1,*]

1 Cavendish Laboratory, University of Cambridge, Cambridge, CB3 0HE, United Kingdom

* pc245@cam.ac.uk

Abstract

This document contains supplementary information on the methods used in the main paper.

S1 File

Section 1 Supplementary Information. **Langevin equation** To simulate the rowers, each rower is updated using the following equation,

$$\dot{\mathbf{r}}_i = \sum_{j=1}^{N} \mathbf{H}_{ij} \cdot (\mathbf{F}(\mathbf{r}_j) + \mathbf{f}_j)\,. \tag{SI1}$$

There are a total of N rowers in the system, which are coupled through the tensor $\mathbf{H}_{ij}$. We used the Blake tensor due to the presence of the wall, the details of which are included in Section 2 Supplementary Information. The value of the driving force $\mathbf{F}$ from the potential depends on the current position of the rower. An illustration of the driving potential is in Figure 2b. At the scales considered, Brownian noise comes into play, represented by the term $\mathbf{f}_i$ [1]. Thermal fluctuations on each bead result from the random forces applied by the fluid molecules. This noise is included using the Ermack-McCammon method to emulate Brownian dynamics [2]. Consequently the mean and variance of the noise are,

$$\langle f_i(t) \rangle = 0, \qquad \langle f_i(t) f_j(t') \rangle = 2 k_B T H_{ij}^{-1} \delta(t - t'). \tag{SI2}$$

The variance depends on the hydrodynamic tensor H_{ij}^{-1} as well as the temperature T and Boltzmann's constant k_B. For an isolated particle, the noise is white with the fluid drag scaling the size of the fluctuations.

Section 2 Supplementary Information. **The Blake tensor.** Near a solid boundary the solution for a point force can be developed using the method of images [3].

The image flows are chosen to ensure the flow at the boundary is zero. The diagonal terms of the tensor, i.e. the drag a bead experiences corrected for the wall's presence, is,

$$\mathbf{H}_{ii} = \frac{1}{6\pi\eta a}\left(\begin{bmatrix}1 & 0 & 0\\0 & 1 & 0\\0 & 0 & 1\end{bmatrix} - \frac{9}{16}\frac{a}{h}\begin{bmatrix}1 & 0 & 0\\0 & 1 & 0\\0 & 0 & 2\end{bmatrix} + h.o.t\right), \tag{SI3}$$

where h is the height above the wall. It has been assumed that the height is constant, i.e. the particle moves in a plane parallel to the wall.

The off-diagonal terms of the Blake tensor are a combination of Stokeslet flows $\mathbf{G}^{Os}$ (Oseen tensors), a stresslet and rotlet $\mathbf{G}^{St}$ (Stokeslet doublet), and a source doublet $\mathbf{G}^{So}$ [4].

$$\mathbf{H}_{ij} = \mathbf{G}^{Os}(\mathbf{r}_i - \mathbf{r}_j) - \mathbf{G}^{Os}(\mathbf{r}_i - \bar{\mathbf{r}}_j) + 2h^2\mathbf{G}^{So}(\mathbf{r}_i - \bar{\mathbf{r}}_j) - 2h\mathbf{G}^{St}(\mathbf{r}_i - \bar{\mathbf{r}}_j). \tag{SI4}$$

The position of the jth rower is $\mathbf{r}_j = (x_j, y_j, h)$. The image rower position is marked by $\bar{\mathbf{r}}_j = (x_j, y_j, -h)$. The expressions for each of the Green's functions are as follows,

$$\mathbf{G}^{Os}(\mathbf{r}) = \frac{1}{8\pi\eta}\frac{1}{r}\left(\mathbf{I} + \frac{\mathbf{r}\otimes\mathbf{r}}{r^2}\right), \tag{SI5}$$

$$\mathbf{G}^{So}_{\lambda\nu}(\mathbf{r}) = \frac{1}{8\pi\eta}\left(1 - 2\delta_{\nu 3}\right)\frac{\partial}{\partial r_\nu}\left(\frac{r_\lambda}{r^3}\right), \tag{SI6}$$

$$\mathbf{G}^{St}_{\lambda\nu}(\mathbf{r}) = \left(1 - 2\delta_{\nu 3}\right)\frac{\partial}{\partial r_\nu} G^{Os}_{\lambda 3}(\mathbf{r}) \tag{SI7}$$

Section 3 Supplementary Information. Dimensionless units. The equation governing the rowers can be made dimensionless. Time and position are expressed in terms of the semi-period τ_0 and oscillation amplitude A, as the oscillations are the defining behaviour for rowers. Stokes' drag coefficient ($\gamma = 6\pi\eta a$) is an appropriate parameter when expressing the tensors we use in a dimensionless manner. The trap force is expressed in terms of the time averaged force $\langle F\rangle_t$, which can also be expressed in terms of the earlier parameters. This results in two dimensionless parameters for the system: the dimensionless noise ξ and velocity parameter $\tilde{V}$. ξ is defined using the spread of the noise, while $\tilde{V}$ is from direct inspection of the Langevin equation. They are defined as,

$$\begin{aligned}\tilde{V} &= \frac{\tau_0\langle F\rangle_t}{A\gamma}, & \xi &= 2k_BT\frac{\gamma}{\tau_0}\left(\frac{\tau_0}{\gamma A}\right)^2\\ &= 1, & &= \frac{2k_BT}{A\langle F\rangle_t},\end{aligned} \tag{SI8}$$

where the relation between time average force with A, γ, and τ_0 has been used to simplify both expressions.

Section 4 Figure Fourier modes to measure chevron frequency.

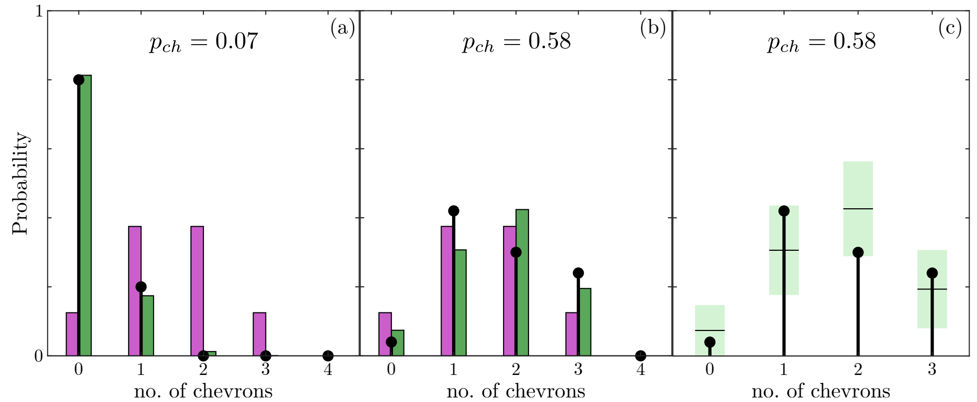

Fig SI1. Details when measuring chevrons and fitting binomials. The number of chevrons are counted using the Fourier terms of the phase profile. (a) An example of a phase profile (black diamonds) with a sine wave overlaid (purple curve) that corresponds to the largest Fourier mode. (b) The associated Fourier modes with the profile in (a). The third mode is clearly dominant, indicating there are three chevrons. (c) A binomial fitting example when $p_{ch} = 0.07$, i.e. the probability is low. The observed data is shown by the black stems, and two theoretical distributions shown in green and purple. The green is the binomial distribution for $p_{ch} = 0.07$, the value measured from simulation, and the purple distribution is the random case where $p_{ch} = 0.5$. (d) An example where the p_{ch} is midway between the extremes of 0 and 1, specifically the probability is measured as $p_{ch} = 0.58$. Again the green distribution has a probability equal to the measured value, and the purple has random chance. The observed values don't align as closely with the theoretical distribution. The variability of binomial distributions depends on p_{ch}, with the peak at $p_{ch} = 0.5$. (e) 95% confidence intervals for observed frequency when $p_{ch} = 0.58$. Every value observed lies within the confidence interval. The ranges are correlated, and the observed values are likely to fall mostly outside the intervals or all within.

References

1. Bruot N, Kotar J, de Lillo F, Cosentino Lagomarsino M, Cicuta P. Driving Potential and Noise Level Determine the Synchronization State of Hydrodynamically Coupled Oscillators. Phys Rev Lett. 2012;109:164103.

2. Ermak DL, McCammon JA. Brownian dynamics with hydrodynamic interactions. J Chem Phys. 1978;69(4):1352–1360.

3. Blake J, Chwang A. Fundamental singularities of viscous flow. J Eng Math. 1974;8(1):23–29.

4. Manghi M, Schlagberger X, Kim YW, Netz RR. Hydrodynamic effects in driven soft matter. Soft Matter. 2006;2:653–668.
