# Supplementary material for: Changes in geometrical aspects of a simple model of cilia synchronization control the dynamical state, a possible mechanism for switching of swimming gaits in microswimmers
Source: PLoS One. 2021 Apr 8;16(4):e0249060. doi: 10.1371/journal.pone.0249060 (PMC8031381; doi:10.1371/journal.pone.0249060)
Supplement: Attachment — Submitted filename: response to referees.pdf [file pone.0249060.s002.pdf]

We thank both reviewers for carefully reading this work and providing very valuable feedback and criticism. We apologise for the delay in addressing this, due in part to the pandemic perturbations. We have made a large number of small changes, in red in the revised text, in response to the reviewer comments, and we addressed all points in detail below.

Reviewer #1: Many microorganism use cilia arrays to propel themselves, for example, by metachronal waves. The authors use the rower model to demonstrate how local changes in its geometrical setting or in the oscillation frequency change the appearance of metachronal waves. They also model the chevron occurrence by a binomial distribution, as they write.

I find the main idea of varying local properties of the rower model in order to hint to gait switching in microswimmers propelled by cilia very attractive. Also, the manuscript in most of its parts is clearly written. However, I have a major comment and a few smaller ones before the manuscript can finally be recommended for publication.

1. The section on ``Consistency of the observed phase profiles'' is pretty incomprehensible. Already in the first paragraph the connection between chevrons, binomial distributions, and Fourier modes should be clearly worked out also by becoming more quantitative. Often the work subset is used but the subset itself is not described. Also why is ``the number of chevrons …. three''.
(page 10, line 233).

So I urge the authors to go over this section and revise it carefully.

We thank the reviewer for encouraging us to improve the introduction and that section of methods. We have now defined “subsets”, and hopefully clarified the methodology.

2. The title is very suggestive but does not really reflect the contents of the article. The authors might think about choosing a more concrete title.

Previous:

Simple geometric controls could allow microswimmers swimming by coordinated ciliary dynamics to switch gaits

A change to:

A simple model for switching of swimming gaits in microswimmers, by geometric control of cilia synchronization.

Emphasises upfront the nature of “model” in this paper.

3. page 3, line 68: … can be switched as a phase transition ….
What does this really mean.

Yes – sentence improved to "We show subsets of cilia can form, with phase-locking in opposing phase, and that these dynamical regimes can be switched (controlled) using the geometry of the system, or small alteration to the oscillators, as control parameters."

4. page 3, line 72: please check the grammar of the second half sentence.
Yes – grammar improved to "The rower model is a phase-free driven oscillator, it represents in a highly simplified fashion the dynamics of cilia \cite{Bruot2016}. Although biological flagella involve shape changes of slender filaments \cite{Elgeti2015,Rosenbaum2002}, each cilium is represented in the model by a single oscillator bead. In the far-field, i.e. for large separation distances, the flow field resulting from this sphere should match closely to that of a filament \cite{Bruot2016}."

5. page 5, lines 127, 128: I did not understand why ``straight sections would not be expected to strongly couple via hydrodynamic forces''. Please explain better.

We have changed that sentence to a clearer statement: "The nature of the solid surfaces, and whether they are flat or curved, also enters the hydrodynamic coupling strength because of screening effects~\cite{Wollin2011}."

Reviewer #2: Last author Cicuta has worked extensively in coupled oscillators with hydrodynamic interactions, and first author Hamilton has likewise published several papers with Cicuta. The simulations are done with the rower model, as in this paper. Quoting the authors: "Although biological flagella involve shape changes of slender filaments [11, 48], in the model each cilia is represented by an oscillator bead. In the far-field, i.e. for large separation distances, the flow field resulting from the sphere should match closely to that of a filament." I have now looked at several papers from the Cicuta group, including some that are aligned with experiments, and some that have just been accepted in 2020. As I read this paper, having worked on the mechanics of transport by coordinated cilia, I sincerely question the relevance of this rower model to hydrodynamic coupling between a few or carpets of beating cilia. I focus solely on viscous fluid coupling, as the authors do in this paper. This is not relevant to coordinated cilia in the lung which Cicuta describes in his Biochemical Society Transactions review submission, nor to flagella propulsion of sperm or bacteria in mucus barriers. There the fluids are mucus, a totally different story. So please make sure to distinguish motile species in viscous fluids as the system you are modeling in an idealized way.

Yes we agree – have now clarified in a few places in the intro and elsewhere that our idealised model is an approximation of purely viscous systems.

In this rower model, the spheres are parallel and move along a 45 degree line. Previous models use parallel 90 degree lines; shouldn't one at least mention the relevance to those results?

We are not completely sure what the reviewer has in mind here. As they say before, our group has been working on this rower model for many years, and we studied the "ground states" of rowers in a variety of geometries and driving conditions, in many papers. We also studied chains of rotors, a related model. We explored transitions from disorder to coupling, the role of thermal noise, complex chimera states… A whole body of those results up to 2016 is outlined in our extensive review, ref [1] in this paper. Here, as we say in the manuscript, we wanted a basic state with strong coupling, possibility of modulating large amplitudes, presence of a solid boundary - but obviously requirement to avoid possible overlapping of spheres. Setting 45 degrees was convenient. We don't think anything qualitatively interesting would change by varying that angle a bit. The key reference on which we build is Wollin-Stark , cited as ref [40].

The neighboring cilia are NEVER far-field, so how is anyone supposed to interpret this model for species motion through beating cilia carpets on a paramecium or a starfish larvae?

We agree that in many of these systems cilia are quite dense. This could have two effects. (1) There could be a steric interaction between the filaments – this we have not attempted to model at all for now, but we note that in Volvox movies there is no evidence of cilia contacts. (2) the hydrodynamics might not fully be in the far field regime. This is true. We have in the past explored the near field of two spheres, and at the level of Rotne-Prager tensors it simply results in changes of the coupling parameters, in a distance-dependent way. This would have the same qualitative effects in our simplistic model as changing slightly the shape of the driving potentials.

The point of the simplistic model is not to be able to make a quantitative prediction that can be ported straight to data from an organism – the value of these models, in our opinion, is to show that control of a complex phenomenon is possible by adjustment of a few simple geometrical properties. What we demonstrate with this model here is that systems of motile cilia, in situations of viscous flow and purely hydrodynamic interaction, have the possibility to exploit the control property we tune in the model. Biology often evolves to make use of robust physical properties, and so we think our simple model might inspire experiments to test if this mechanism is at play in some biological systems.

We have tuned some of the sentences in the conclusion to reflect this.

Furthermore, Blakeslets and resistive force theory from oscillating spheres are not a good approximation of slender-body-induced fluid flow except for a straight filament. Now you have fields of titled rowers, with a square root potential that appears to come from out of the blue. Why power law 1/2? How sensitive are the results to this seemingly arbitrary choice?

The importance of the shape of the potential (scanning potentials with different curvatures) was explored by us in [Driving potential and noise level determine the synchronization state

of hydrodynamically coupled oscillators Physical Review Letters 109, 164103 (2012)], ref 39 in the manuscript. There we showed that a linear driving potential gives no coupling; as the power law of the potential is increased or decreased from unity, there is increased coupling. Neighboring spheres will settle in anti-phase for potentials with power>1, and in-phase for potentials with power<1. We chose here the power 0.5 but could have chosen anything between 0 and unity. Changing the power (on either side of unity) just re-scales all the coupling strengths.

You then study the effects due to 3 control parameters: rower density, amplitude and frequency. Then, there is a big focus in the simulation results on chevrons. Where, if ever, have chevrons been identified in motile microscopic species, either synthetic or natural? Since the results are centered around chevrons, this seems a must to connect the model predictions with observations. No references mention chevrons, nor are any experimental observations mentioned (that I can find). Is this a prediction you are making with experiments to be performed?

We wanted to be careful not to imply, lacking direct evidence, that we had the explanation for gait switches in organisms that are potentially quite complex such as starfish larvae, see ref. 27. Those, and the well documented gaits in Paramecium, were the data that inspired us.

We had failed to explain the connection between "chevrons" and the sorts of data and videos that we are used to seeing in e.g. ref 27. This is now added at the beginning of "results", where the term chevron is first presented.

I am both impressed and disappointed in this paper. It is a significant study, clearly based on the synthesis of many simulations. However, I am really not at all convinced that even the qualitative observations across the 3 control parameters have anything to do with biology or synthetic experiments. This leaves me conflicted: nice idealized modeling and simulations and cool phenomenological results, but the putative relevance to propulsive cilia coordination of microswimmers to switch gaits (an evolutionary necessity) is hard to swallow.
If the authors were forthcoming with respect to any evidence for relevance to microswimmers, I am in favor of publication. If the authors presented experimental validation of these predictions, my opinion would be overwhelmingly positive, and I would gladly eat the criticisms. Such a result would mean that this extremely idealized model of cilia-induced hydrodynamics is a valuable qualitative tool. Given the large body of work on rowers, experimental validation is necessary.

We fully accept that this paper is "putative" – in the sense that we are not demonstrating that our model (itself, hopefully, robust!) is modelling a specific biological system. As explained above however, it is quite possible that some biological systems will be exploiting the physics demonstrated in this model. We hope that this is now clearer in the manuscript, having improved connection between the chevron states and the starfish larvae observations, and other small changes like the addition of new reference [4] to another class of swimmers with metachronal waves.
