# Supplementary material for: Changes in geometrical aspects of a simple model of cilia synchronization control the dynamical state, a possible mechanism for switching of swimming gaits in microswimmers
Source: PLoS One. 2021 Apr 8;16(4):e0249060. doi: 10.1371/journal.pone.0249060 (PMC8031381; doi:10.1371/journal.pone.0249060)
Supplement: Attachment — Submitted filename: response to referees_jan21.pdf [file pone.0249060.s003.pdf]

Dear Editor,

I'd like to thank both referees for their time and expertise on this manuscript. Reviewer # 1 was satisfied by the first round of review. Newly involved Reviewer #2 has a number of comments, most of which are extremely valid, but some of which require substantial further work which we are now unfortunately unable to carry out. The main problem is that the PhD student active on this work graduated in June 2019. We submitted to PLOS One in December 2019, and since then the student has moved to a non-academic job in Australia and has lost the possibility to run new simulations. Nothing wrong with the supervisor taking over, which I did already for the October 2020 revision, but there's a limit to how much original research I am able to put into this. Anyway – I am doing my best again also on this revision, and reviewer # 2 does kindly offer various options as to how to address their points.

Best regards,

Pietro Cicuta

Reviewer #1: The authors responded to all comments. I can now recommend the manuscript for publication.

Reviewer #2: This article proposed a theoretical model for metachronal waves on the surface of ciliated organisms. The model is an extension of a versatile and insightful rower model employed by Cicuta and collaborators, in a number of recent publications. The suggestion that organisms can possibly control the phase delay between nearby cilia and the nature of metachronal waves by changing certain physical parameters associated with ciliary arrays is indeed an intriguing and sensible idea.

However, the present model itself is highly idealised, both in terms of the dynamics of the individual rowers, and in their arrangement in a linear one-dimensional chain with wrapped boundary conditions. I believe additional work will be required to extend this to make it biologically relevant. The authors did not attempt to connect the synchronization patterns they obtained to swimming dynamics, and provided no evidence (either theoretical or experimental) to suggest that transitions in the patterns of such oscillators can actually reverse or significantly alter the fluid landscape around the ciliated swimmer. The association with 'swimming gaits' in real organisms is at best tenuous, and at worst misleading.

I completely agree that the model is extremely idealised. Indeed, because they do not have the cycle-dependent change of drag coefficient which is thought to be a key property of cilia in their power and recover strokes., these models are so idealised that it does not make sense to look at the details of flow patterns (i.e. the propulsion) for these active bead systems. The bead chains can still cause propulsion (by sustaining a travelling wave, the extension of a 3-bead swimmer we

investigated experimentally ourselves in 2010) but for an even vaguely quantitative connection to flows and propulsion in cilia I think the drag change in power/recovery would be dominant.

The value of these *extremely* simplified models is really to highlight that a certain emergent, complex, collective behaviour can arise from minimal elements. Then, in the minimal model, one can play some qualitative changes against each other (here, the geometry, coupling and modulation of forcing along a chain). This is now much more explicit in abstract and introduction.

In my view the authors have two options towards publication, either revise the paper to make it clear this is a theoretical model looking at synchronization phenomena in a 1D chain of oscillators designed to mimic cilia (i.e. do not refer to 'swimming gaits' in the title, tone down the claims of abstract – which are not supported by the results of their current model, and reserve all discussion of how they think this could translate in real organisms to the discussions section, which they could expand to include free-swimming), or else provide further experimental or theoretical evidence that 'swimming' will be affected by the types of perturbations considered in this work.

Given the history of this work, and my remark above, I have to take the "toning down" angle of this suggestion. I have changed the title, from:

"A simple model for switching of swimming gaits in microswimmers, by geometric control of cilia synchronization"

To:

"Geometrical aspects of a simple model of cilia synchronization control the dynamical state, a possible mechanism for switching of swimming gaits in microswimmers",

which I think is factually correct.

I have completely rewritten the abstract, edited the introduction significantly, and in various places including conclusions I have further qualified the putative nature of the connection to living systems.

key comments.

1. *Stronger justification for why the rower models 'are expected to produce general synchronisation features in viscously coupled systems'. This is a very general statement. The authors should explain how the rower model performs compared to other commonly used cilia models, and what are its advantages and limitations.*

Maybe the phrase was unclear. I have changed it. The point is correct though: both rower and rotor models (which are the two commonly used models to look at synchronisation of cilia; we have studied both over the years from theory and experimental perspectives) generally synchronise with the exception of "singular" pathological choices of parameters. Our systematic review in 2016 [ref 1] covers everything that was known on rower models up to that point. The major discovery since then has been to show that an effective-phase-coupling description can be written for rowers [ref 44] as had been done on rotors by Uchida and Golestanian. Given that the mathematics of the coarse-grained models is now unified, the differences between the classes of models are in the details. Rowers still offer a strong theoretical description based on hydrodynamic modes. Rotors

have an advantage of describing more directly the possibly complex trajectories of the center of drag of a filament.

I have changed the sentence highlighted by the referee, and added some of these considerations to the last paragraph of the introduction section.

2. *The authors consider the dynamics of a chain of rowers – but this is far from a 2D carpet or a ciliated surface. Why should this very idealised geometry be expected to represent what happens in the cell? Why the choice of 45 degree incline – seems arbitray, what is the motivation for this set-up?*

I have clarified that 45 degrees is somewhat arbitrary: we wanted an angle that would allow variations in the stroke amplitudes without leading to overlaps of the point forces or even approaching near field.

If we imagine a variety of organisms (like Volvox, starfish larvae and others) that have a cylindrical symmetry, then the 1d chain can be thought of as one line of "longitude". I have clarified this.

3. *Another point about choice of parameters. This is not well explained – for different organisms exhibit very different metachronal wave properties, differ in cilia length, spacing between cilia etc. The authors appear to be inconsistent throughout about which class of organisms their work is modeling – they mention simulation parameters inspired by starfish larvae, yet the drawings and discussions focus on paramecium (much smaller scale)? Then in the conclusions they discuss corals? Their work is clearly more relevant for singular rings of cilia (found in some organisms), rather than sheets.*

Given the ideal nature of the model system, choice of parameters is not tied to a particular organism. Clearly complex organisms can have modulation connected to a neural system, whereas single cell organisms like paramecium presumably expose all the cilia to a quite uniform chemical environment. However in all ciliated organisms, in principle, there can be effects of geometry and modulation of activity such as the ones we idealise here. I think the domain where this is most interesting is sheets of cilia, with a symmetry (e.g. the many axial organisms) that means we can look at 1d. I have elaborated on this in the introduction and in the materials and methods.

4. *According to the operational principle of most cilia, beat frequency and amplitude will be strongly coupled – it's unlikely that one will be varying but not the other. Therefore distinguishing between control mechanisms b & c seems to be somewhat problematic? Can this be better justified? (I refer back to 1.)*

In the model, we can obviously control frequency and amplitude separately. I agree that in a real system (physical or biological!) we would expect the two to be correlated, if anything out of conservation of energy dissipation. But there's no fundamental reason that they should be, say, exactly inversely correlated. We don't have a complete picture of what sets the waveform on a cilium, so I don't think we know to what extent a given cilium could be made to modulate amplitude and frequency separately. These would also depend on external factors, such as the drag felt by the filament. I think it's potentially useful to explore the two parameters separately, in the model system where this is possible.

5. *Are the authors aware of any examples of metachronal wave reversal in real organisms induced by a change in separation between some of the cilia? They mention the starfish larvae example – but it is unclear if those patterns have anything to do with shape changes? Or frequency? What about this idea that some level of disorder in the spacing could help with control the generation of locally synchronized subsets (lines 306/307) – is there any evidence from the literature? (Note that the well-documented gaits in paramecium are unlikely to result from a mechanism such as described by the present model. In paramecium, and no doubt other species, the switch occurs due to some rapid cellular signalling, which abruptly halts or reverses the direction of ciliary beating.)*

In the starfish larva (the videos of the papers cited) it seems to me that there is a clear shape change together with the change of cilia dynamical state. That was the observation that motivated me in the direction of this paper. I am not aware of experimental evidence that these mechanisms are at play, hence the toning down of the title and other claims in abstract and conclusions. However, nobody presumably has looked, and hopefully the physical possibility of the proposed mechanisms here can lead to testing out these conditions. Nature/evolution have a knack of exploiting physical mechanisms where they exist, since they come “for free” and can be quite robust.

6. *The analysis of the chevron profiles using statistical methods – though interesting, it is unclear how this would relate to swimming. Are the authors suggesting that phase reversals are associated with reversals in the actual swimming direction?*

Yes, the phase reversals would correspond to a change in swimming velocity, or direction, or a change in the patterns of flow to affect foraging. I have added this sentence.

7. *One suggestion would be to compare flow pumping by these chains of rowers – this would still be far from a force/torque free swimmer, but would be more relevant to biological systems. See for example https://www.pnas.org/content/117/48/30201.short.*

We were not aware of this paper from December 2020, two months after our resubmission. We are citing it now. As mentioned in my first response to the general comments, and then at point 3, this model as it is (our drag is the same in power and recovery strokes) cannot sensibly be used to argue about flow fields. One would need to have single-cilium kinematics on a given system (so a particular biological system would have to be chosen for which such data exists), then include that into the simulation (either as explicit filaments, or as a hard-coded change in effective drag of the Oseen sphere, the point force). Nobody has done either of these things systematically in the context of cilia synchronization, and it is beyond the scope of this paper (let alone our current capability).

8. *There are many ways to control swimming gait using cilia, hydrodynamics is just one possibility. In fact, many more authors have studied non-hydrodynamic mechanisms for changing the swimming trajectory, these should be discussed. See for example studies on paramecium escape reactions, or even the steering gaits of uniflagellate sperm which are example of shape changes.*

The sperm example, I would argue, confirms with a single-cilium case that changes in the waveform of the cilium are important in the context of gaits. In our model, all the cilium properties are reduced to how a bead is driven. I think this is described quite extensively in the introduction, and it is a point we have made in numerous previous papers. As to non-hydrodynamical possibilities, yes I have added more "warnings". But the point of this model-based paper is precisely to hypothesise that a physical (simpler? More robust?) mechanism might be at play in some of the examples where gait-change has been observed. This is not impossible, and I do not know of a "complete" and validated theory of how the single-cell paramecium actually does gait-change. Why could it not be a result of switching between two metastable dynamical states, a switch that can be triggered from a mechanical perturbation?

Other:

Line 41: Ciliophora is not a genus, and the term is being phased out

Thanks - I have removed this term.

Line 43: Central nervous system – missing word

Thanks – fixed.

Fig 1 - what is the purpose of the green/pink color scheme? This could be confusing as panels of the same color don't match up.

Sorry – that was cosmetic only, I have now made panels uniform.

Fig 2- panel c - the drawing does not really make it clear all the rowers are on the same plane, and that this plane is above a wall. Make the plane more 3d?

Not sure how to make it more 3d, I have checked the caption and it's quite complete. I have added "isometric view" next to the label (c).

Fig 4 – what is Ng here, presumably 10? This should be stated explicitly.

Yes, N_g =10, this is now specified.
